# 2b-RAD Genotyping of the Seagrass *Cymodocea nodosa* Along a Latitudinal Cline Identifies Candidate Genes for Environmental Adaptation

**DOI:** 10.3389/fgene.2022.866758

**Published:** 2022-05-16

**Authors:** Miriam Ruocco, Marlene Jahnke, João Silva, Gabriele Procaccini, Emanuela Dattolo

**Affiliations:** ^1^ Stazione Zoologica Anton Dohrn, Naples, Italy; ^2^ Department of Marine Sciences, Tjärnö Marine Laboratory, University of Gothenburg, Gothenburg, Sweden; ^3^ Centre of Marine Sciences, University of Algarve, Faro, Portugal

**Keywords:** seagrass, SNPs, 2b-RAD, outlier loci, latitude, flowering

## Abstract

Plant populations distributed along broad latitudinal gradients often show patterns of clinal variation in genotype and phenotype. Differences in photoperiod and temperature cues across latitudes influence major phenological events, such as timing of flowering or seed dormancy. Here, we used an array of 4,941 SNPs derived from 2b-RAD genotyping to characterize population differentiation and levels of genetic and genotypic diversity of three populations of the seagrass *Cymodocea nodosa* along a latitudinal gradient extending across the Atlantic-Mediterranean boundary (i.e., Gran Canaria—Canary Islands, Faro—Portugal, and Ebro Delta—Spain). Our main goal was to search for potential outlier loci that could underlie adaptive differentiation of populations across the latitudinal distribution of the species. We hypothesized that such polymorphisms could be related to variation in photoperiod-temperature regime occurring across latitudes. The three populations were clearly differentiated and exhibited diverse levels of clonality and genetic diversity. *Cymodocea nodosa* from the Mediterranean displayed the highest genotypic richness, while the Portuguese population had the highest clonality values. Gran Canaria exhibited the lowest genetic diversity (as observed heterozygosity). Nine SNPs were reliably identified as outliers across the three sites by two different methods (i.e., BayeScan and pcadapt), and three SNPs could be associated to specific protein-coding genes by screening available *C. nodosa* transcriptomes. Two SNPs-carrying contigs encoded for transcription factors, while the other one encoded for an enzyme specifically involved in the regulation of flowering time, namely *Lysine-specific histone demethylase 1 homolog 2*. When analyzing biological processes enriched within the whole dataset of outlier SNPs identified by at least one method, “regulation of transcription” and “signalling” were among the most represented. Our results highlight the fundamental importance signal integration and gene-regulatory networks, as well as epigenetic regulation *via* DNA (de)methylation, could have for enabling adaptation of seagrass populations along environmental gradients.

## Introduction

Environmental heterogeneity in space and time can impose a strong selective pressure driving adaptive divergence of populations (Linhart and Grant, 1996; Bergland et al., 2016; Urban et al., 2020). Quantifying the extent of this differentiation and identifying loci underlying such divergence is a major aim of evolutionary and ecological genetics (Feder and Mitchell-Olds, 2003; Savolainen et al., 2013) and seascape genomics (Selkoe et al., 2016). Environmental gradients, where environmental factors change along a geographic scale, offer a great opportunity for understanding patterns and processes responsible for phenotypic changes of populations (Endler, 2020). There are examples from a variety of species, where phenotypes tend to change in predictable ways across large-scale gradients such as latitude, altitude, or water depth (Endler, 2020). These geographical patterns can reflect genetic variation or phenotypic plasticity and eventually represent adaptive variation in response to selection gradients (Conover et al., 2009; Sanford and Kelly, 2011; Schneider and Meyer, 2017).

Variation in specific environmental variables (e.g., temperature or photoperiod) across geographical clines is directly related to aspects of phenotypic and genetic divergence among populations in land plants (Linhart and Grant, 1996; Hut and Beersma, 2011; Hut et al., 2013). For instance, climate is one of the most important drivers of adaptive phenotypic traits in forest trees (Richardson et al., 2009; Eckert et al., 2010). Fitness-related traits such as survival, growth and biomass partitioning have been shown to vary along temperature gradients associated with altitudinal and latitudinal clines or according to different precipitation and aridity regimes (Aitken and Hannerz, 2001; St Clair et al., 2005). Similarly, other traits associated with major life history events, e.g., seed dormancy and flowering time, were found to follow a clear latitudinal pattern in *Arabidopsis thaliana* accessions collected throughout the European range of the species (Debieu et al., 2013). In particular, timing of flowering across accessions was linked to molecular polymorphisms in key regulatory genes that control this trait, such as FRIGIDA and FLOWERING LOCUS C (Le Corre et al., 2002; Stinchcombe et al., 2004; Shindo et al., 2005).

In marine plants, these studies are still hampered due to the absence of genomic resources for most species (with few exceptions e.g., *Zostera marina*) and the general unfeasibility to perform molecular genetic studies across multiple generations. The reproduction of seagrasses under controlled conditions is indeed still challenging, and life cycles are often too long, which prevent identifying heritable variations (Hu et al., 2020; Pazzaglia et al., 2021). Seagrasses are the only group of flowering plants that has returned to the sea, (re)adapting their physiology and morphology to a completely submerged lifestyle (Wissler et al., 2011; Golicz et al., 2015; Lee et al., 2016; Olsen et al., 2016; Lee et al., 2018). Despite their low taxonomical diversity, they successfully colonized most coastal shores worldwide (Spalding, 2003; Larkum et al., 2006), providing fundamental ecosystem services (Ruiz-Frau et al., 2017) and contributing to climate change mitigation (Marbà et al., 2015; Stankovic et al., 2021). The current distribution of seagrass species along geographical and depth gradients is influenced by their tolerance window for environmental drivers, such as light, temperature and salinity (Short et al., 2007).

To date, population genetic studies in seagrasses have mostly relied on a limited number of nuclear DNA markers (e.g., simple sequence repeats, SSRs). Such studies have aided in resolving main geographic differentiation and structure of seagrass populations (e.g., Alberto et al., 2006; Arnaud-Haond et al., 2007; Alberto et al., 2008; Serra et al., 2010; Hernawan et al., 2017; Bijak et al., 2018; Jackson et al., 2021). Indices of genetic diversity based on SSRs have also been correlated with local environmental disturbances (Jahnke et al., 2015b), fundamental plant traits such as flowering synchronization (Jahnke et al., 2015a) and ecosystem functioning and resilience (Reusch et al., 2005; Hughes and Stachowicz, 2011). In a few studies, molecular polymorphisms have been functionally characterized through genome scan approaches and related to contrasting habitat types, depth and latitudinal gradients (Oetjen and Reusch, 2007; Oetjen et al., 2010; Jahnke et al., 2019). Yet, gene expression studies employing common-garden or reciprocal transplantation approaches have collectively suggested local adaptation of seagrass populations to thermal or light gradients (Franssen et al., 2011; Winters et al., 2011; Franssen et al., 2014; Jueterbock et al., 2016; Dattolo et al., 2017), even if the heritability of the observed phenotypic differences remain elusive.

*Cymodocea nodosa* (Ucria) Ascherson is a marine dioecious angiosperm present throughout the Mediterranean Sea and the adjoining Atlantic coasts and the dominant species structuring subtidal seagrass ecosystems along North West Africa and South West Europe (Green and Short, 2003; Alberto et al., 2008). Recent species distribution models predict the most relevant environmental variables defining its distribution to be sea surface temperature (SST) and salinity (Chefaoui et al., 2016). Large inter-regional and local-scale variations in abundance and structure (i.e., morphology and biomass allocation), as well as the extant of sexual reproduction (as seed production) in *C. nodosa* have been described and correlated with different environmental conditions [i.e., seasonal patterns of Photosynthetically Active Radiation (PAR) and SST], and evolutionary contexts (i.e., genetic diversity) (Máñez-Crespo et al., 2020). Populations inhabiting distinct biogeographical regions have also been shown to possess a differential resilience, performance and recovery capacity under local perturbations (e.g., shading) (Tuya et al., 2019; Tuya et al., 2021). The higher sensitivity of certain meadows to disturbance, as those at the peripheral distribution of the species in the Canary Islands, has been attributed to their genetic isolation and low genetic diversity (Tuya et al., 2019; Tuya et al., 2021). An important geographic heterogeneity was also observed in *C. nodosa* plants from contrasting thermal environments (warm-adapted vs. cold-adapted plants) in response to heatwaves, in terms of diversion of carbon reserves and biomass allocation (Marín-Guirao et al., 2018). However, molecular studies addressing how environmental gradients could affect local adaptation of natural populations through adaptive genetic variation at specific loci are currently missing in this species.

Restriction-site-associated DNA sequencing (RAD-Seq) techniques represent a family of cost-effective techniques compared to e.g., whole-genome sequencing that can be employed in non-model species without a reference genome and guarantee high-resolution population genomics data for demographic analyses (Andrews et al., 2016; Shafer et al., 2017). In addition, these approaches offer better opportunities in respect to those based on other markers (e.g., SSRs) to identify loci with a putative signal of selection from the background of neutral variation, and to test their functional importance by associating nucleotide variation in these genes with phenotypic variation in adaptive traits in natural populations (Storz, 2005; Biswas and Akey, 2006). RAD-seq approaches have been applied in marine engineering species (e.g., kelp, Guzinski et al., 2020; Fucales, Reynes et al., 2021a or Mediterranean corals, Pratlong et al., 2021) shedding first light on the detection of candidate SNPs for local adaptation of populations.

**FIGURE 1:**
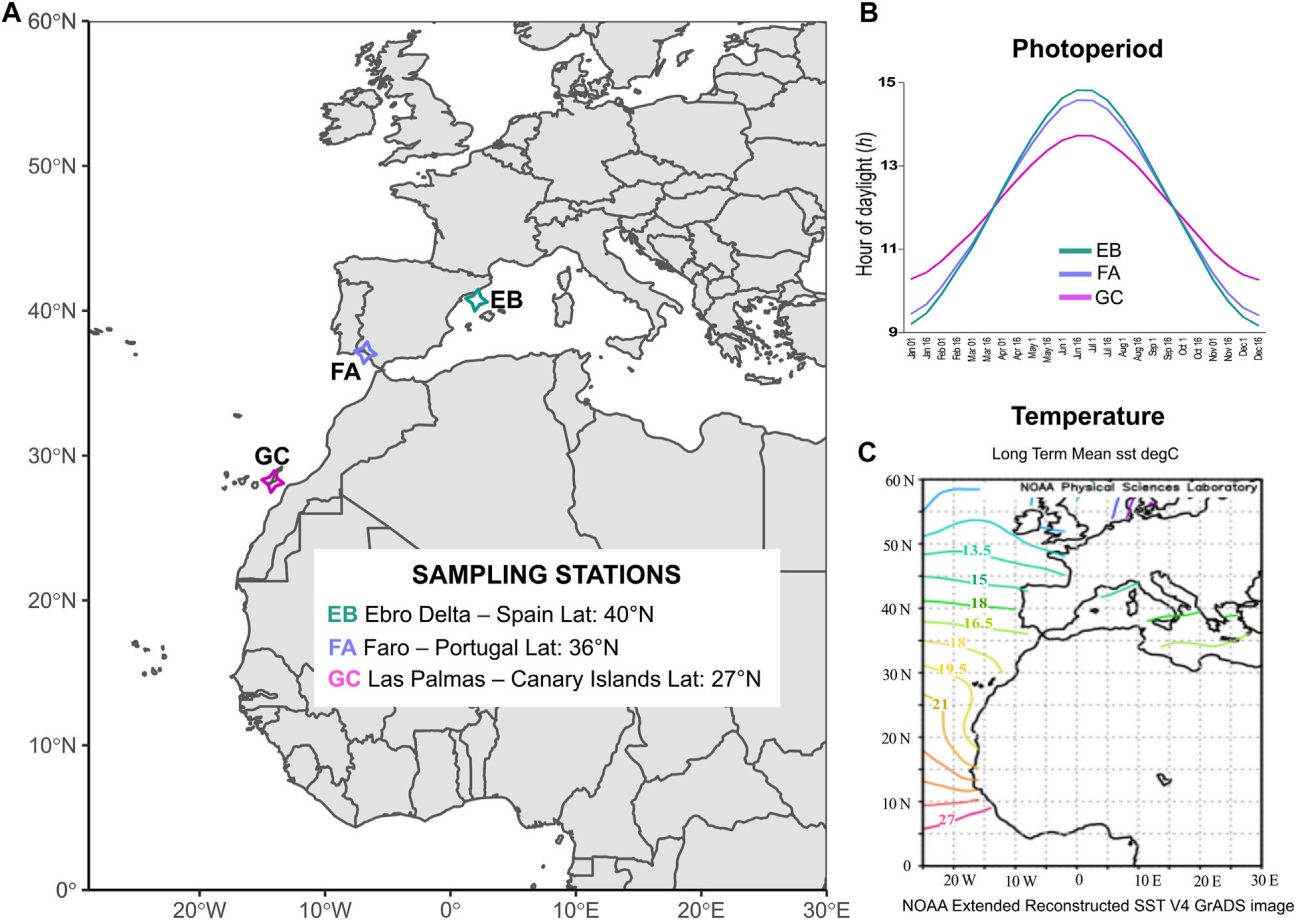
Sampling scheme and environmental characteristics of the selected sites. **(A)** Sampling locations of *C. nodosa*: GC—Las Palmas de Gran Canaria (Lat: 27°N, Canary Islands); FA—Faro (Lat: 36°N, Portugal); EB—Ebro Delta (Lat: 40°N, Spain). **(B)** Annual patterns of photoperiod for the different sampling sites. Daylight hours were derived from annual data and averaged for every 15 days (https://www.timeanddate.com). **(C)** Long term monthly means of Sea Surface Temperature (SST) registered on the Atlantic and Mediterranean waters from NOAA derived from data for years 1971–2000 (https://psl.noaa.gov/data/gridded/data.noaa.ersst.v4.html).

In the present study, we used 2b-RAD genotyping (Wang et al., 2012) to characterize population differentiation and levels of genetic diversity among three populations of *C. nodosa* distributed along the Atlantic-Mediterranean transition region (i.e., Gran Canaria and Faro in the Atlantic and Ebro Delta in the Mediterranean; Figure 1A). Our main aim was to search for outlier loci that could underlie adaptive differentiation of populations across the latitudinal distribution of the species. The underlying hypothesis was that such genetic polymorphisms could be associated to local adaptation of populations to varying photoperiod-temperature regimes across latitudes. We also used SNP markers for assessing the clonality level of populations and compared these data, together with genetic diversity and differentiation, with estimates based on 7 SSR markers previously developed for the species.

## Materials and Methods

### Study Sites and Sample Collection

Individual shoots of *Cymodocea nodosa* were collected at three locations across the Atlantic-Mediterranean boundary from shallow-water meadows (1–4 m depth), thus encompassing most of the latitudinal distribution range of the species (Alberto et al., 2006; Cunha and Araújo, 2009). Collection sites were selected based on their geographic position along a latitudinal gradient and for sampling feasibility. Two sampling stations were located in the north-eastern Atlantic Ocean, i.e., Las Palmas de Gran Canaria (El pajar) (GC)—Canary Islands, Spain (27°45′14″N, 15°40′18″W) and in the Ria Formosa lagoon (Ilha da Culatra) (FA)—Faro, Portugal (36°59′23″N, 7°50′01″W), while the latter was located in the western Mediterranean Sea, i.e., Ebro Delta (EB)—Spain (40°34′48″N, 0°35′45″E) (Figure 1A). The Canary Islands represent the western distributional limit of the species, and are close to its southern distributional limit in Banc d’Arguin in Mauritania (Green and Short, 2003). The Ebro population represents the northernmost sampling site in our study, although the species extends up to the north Adriatic Sea (Venice lagoon, 45.60°N) (Rismondo et al., 2003; Sfriso and Facca, 2007).

In Gran Canaria, daylight hours oscillate between 10 h d^−1^–14 h d^−1^ throughout the year (Figure 1B). In the other two sites, there are greater fluctuations of daylight during seasons: the minimum is ca. 9 h d^−1^ in winter, while the maximum is 15 h d^−1^ during the summer solstice (Figure 1B). In Gran Canaria, the average seawater temperature (SST) during the year is about 19°C (Figure 1C). Faro, the coldest of our sites, displays an average SST during the year of around 16.5°C while Ebro Delta in the Mediterranean, despite its northern latitude, has an average seawater temperature of 18°C (Figure 1C). Twenty *C. nodosa* shoots were collected by snorkeling at each site at a reciprocal distance of around 3–5 m to minimize the risk of sampling within the same clonal patch (Arnaud-Haond et al., 2007; Serra et al., 2010; Jahnke et al., 2017; Jahnke et al., 2019). This number of samples can be considered representative of the meadows and it is comparable to what generally used in 2b-RAD studies (e.g., De Wit et al., 2020; Jahnke et al., 2022). Subsequently, leaf material (about 5–7 cm) was carefully cleaned from epiphytes and dried in silica gel prior to DNA isolation.

### 2b-RAD Genotyping

Genomic DNA was extracted using the NucleoSpin® 96 Plant II kit (Macherey-Nagel) following the manufacturer’s instructions. DNA quality and quantity were checked through 1% agarose gel electrophoresis, and the Qubit^TM^ dsDNA BR assay kit (Thermo Fisher Scientific). 2b-RAD libraries were prepared following a modification of the protocol described by Wang et al., 2012 and available at https://github.com/z0on/2bRAD_denovo. Briefly, genomic DNA (∼100 ng) was digested using the type 2b restriction enzyme BcgI to produce uniform fragments of 36-bp, to which adaptors were ligated on the cohesive ends. The fragments were then amplified with barcoded adaptors and purification of the target bands was carried out by 2% agarose gel-electrophoresis. Subsequently, gel fragments were cleaned using a MinElute Gel Extraction Cleaning Kit (Qiagen) and pooled equimolarly into a single pool. Four individuals from each locality were used as technical replicates (i.e., replicated library preparation and sequencing). These technical replicates were used in the analysis step to assess the overall accuracy of genotyping, set quality filtering criteria, and quantify error rates between samples. In total, 70 *C. nodosa* samples (20 individuals +4 technical replicates for EB and FA; 18 individuals +4 technical replicates for GC) were successfully sequenced on two lanes of an Illumina NovaSeq platform, generating 50-bp paired-end reads, at the Science for Life Laboratory (SciLifeLab) Genomics, SNP&SEQ Technology Platform in Uppsala University, Sweden. Only reads 1 (“forward reads”) were used for this analysis.

Bioinformatic analyses were performed using the computer cluster of the Bioinforma Service of Stazione Zoologica Anton Dohrn (SZN), Naples (Italy). As no genome sequence is currently available for *C. nodosa*, the analysis followed a modified *de novo* pipeline available at https://github.com/z0on/2bRAD_denovo. Reads were first ‘demultiplexed’ based on barcodes and then adaptors were removed, then a quality filtering was performed using the Fastx-toolkit (Gordon and Hannon, 2010). Only reads containing 100% bases with a PHRED quality score of at least 20 were retained for downstream analysis. After trimming and quality filtering, we obtained a total of 82,883,209 reads. Individually trimmed fastq files were then merged to collect tags found in at least two individuals with a minimum depth of five for genotyping. Reads that had more than seven observations without reverse-complement were removed. Subsequently, tags were clustered with CD-hit (Li and Godzik, 2006) allowing for up to three mismatches, followed by the creation of a “reads-derived reference genome” based on 30 fake chromosomes, on which individual trimmed fastq files were mapped back using Bowtie 2 aligner (Langmead et al., 2009).

SNP-calling was performed using The Genome Analysis Toolkit (GATK) version 3.8 (McKenna et al., 2010). A first round of putative variants was generated using GATK’s UnifiedGenotyper, followed by base quality score recalibration (BQSR/BaseRecalibrator and PrintReads) based on a high confidence (>75th quality percentile) SNPs dataset. The realigned and recalibrated reads were then used to perform a second round of UnifiedGenotyper. We used the variant quality score recalibration (VQSR) step to generate an adaptive error model using the SNPs that were consistently genotyped across the technical replicates. A further filtration step was performed with vcftools (Danecek et al., 2011) to remove poorly-genotyped samples and to select only biallelic loci genotyped in at least 90% of individuals, and with a maximum heterozygosity of 50%. Harsh genotyping rate cut-off is recommended for best quality and to avoid RAD loci affected by null alleles because of mutations in the restriction site.

### Population Genomic Analyses

Our final dataset was thinned in order to keep one SNP per RAD fragment with maximal allele frequency (script thinner. pl with criterion = maxAF; https://github.com/z0on/2bRAD_denovo). Technical replicates were discarded from further analyses after examination for concordance in genotype estimates. Individual genetic variation was explored by a Principal Component Analysis (PCA) using the R package adegenet v. 2.1.3 (Jombart, 2008) and by using ADMIXTURE 1.3.0 (Alexander and Lange, 2011). To choose the best estimate of number of clusters (K), we used the ADMIXTURE’s cross-validation procedure with default settings. The hypothetical number of K was set from 1 to 15. PCA and ADMIXTURE analyses were re-calculated after removal of outlier loci confirmed by both employed approaches in the dataset (see 2.4), in order to determine whether outliers had disproportionate or distortive effects on the genetic structure analysis. As the analysis with all loci and only neutral loci showed similar patterns (data not shown), we kept all loci in the analyses. Significance levels of genic differentiation for each population pair (exact G test) across all loci was calculated with GENEPOP 4.7.5 (Rousset, 2008) with default Markov chain parameters (Dememorisation = 10000, Batches = 100, Iterations per batch = 5000). Pair-wise Weir and Cockerham mean and weighted F_ST_ estimates between *C. nodosa* populations were calculated with vcftools (Danecek et al., 2011). Observed heterozygosity (H_o_) as well as F_IS_ across all loci for each population were calculated using the R package diveRsity 1.9.90 (Keenan et al., 2013). The number of distinct Multi Locus Lineages (MLLs) for each population was calculated using the R package poppr (Kamvar et al., 2014; Kamvar et al., 2015). The genetic distance limit for setting delimitation of clones (Hamming distance >0.073) was determined based on the maximum genetic distance detected between technical replicates (i.e., “confirmed clones”). The genetic distance tree of individual samples obtained with poppr is depicted in Supplementary Figure S1. After clone delimitation, F_ST_, F_IS,_ and heterozygosity (H_o_) were recalculated keeping only distinct MLLs per population.

Genetic diversity and differentiation, as well as clonal diversity results were compared with those obtained for the same populations using 7 highly polymorphic SSR markers (microsatellites) developed for *C. nodosa* (Ruggiero et al., 2004; Supplementary Table S1) and used in previous population genetics studies (Ruggiero et al., 2005a and b; Tuya et al., 2019). Only a subset of samples could be genotyped with both techniques (12 individuals for GC; 18 for FA and 12 for EB). Multiplex PCR amplifications were conducted in 25 μL reaction volumes containing 12.5 μL QIAGEN Multiplex PCR Master Mix (QIAGEN), and 0.5 μL DNA (6–10 ng). Thermal cycling consisted of 95°C for 15′, 35 cycles of 94°C for 60″, 58°C for 90″, and 72°C for 90″, followed by 72°C for 30'. PCR products were analyzed on an Automated Capillary Electrophoresis Sequencer 3730 DNA Analyzer (Applied Biosystems). Linkage disequilibrium (LD) and deviations from Hardy-Weinberg equilibrium (HWE) at each locus and across all loci in each population were tested with Genepop 4.7.5 (Rousset, 2008), using 1000 dememorisations, 100 batches and 1000 iterations per batch. LD was not detected among loci, indicating they behaved independently. The number of MLGs was identified using the GIMLET software (Valière, 2002). Clonal diversity was calculated as the *R* ratio: *R* = G-1/N-1, where G is the number of genotypes and N is the number of samples (Dorken and Eckert, 2001). Observed heterozygosity and inbreeding coefficient, as well as pairwise F_ST_ between populations were obtained with GenAlEx 6.503 (Peakall and Smouse, 2012).

### Outlier Loci Identification and Functional Annotation

To identify putative loci under selection, we used two statistical tools employing two different approaches based on F_ST_ and PCA, respectively, i.e., BayeScan v. 2.1 (Foll and Gaggiotti, 2008) and pcadapt v. 4 (Luu et al., 2017; Privé et al., 2020). BayeScan estimates F_ST_ for each SNP locus to perform a genomic scan for outlier F_ST_ values through a Bayesian method. It was used with prior odds set to 100, using thresholds of q = 0.3 and posterior probability *P* > 0.7 (Foll and Gaggiotti, 2008). The pcadapt method tests how much each variant is associated with population structure, assuming that outlier variants are indicative of local adaptation. Based on the scree plot (Jackson, 1993), we set 2 as the best number of principal components, and used the Manhattan plot and Histogram of *p*-values to graphically examine the presence of outliers. Significant outliers were then determined using the Bonferroni correction method for multiple comparisons with the R function p. adjust and α = 0.001. The outputs from the two methods were compared for overlap and shared loci were considered as the “best outliers”. Allele frequency of shared outlier loci was determined with GENEPOP 4.7.5 (Rousset, 2008). Outlier identification was repeated after removal of clone samples from the dataset (i.e., keeping only distinct MLLs for each population).

To determine if shared and non-shared outlier loci could be included in a potential coding sequence, chromosome regions of a length of 80 bp, corresponding to two adjacent 2b-RAD tags around each SNP of interest, were mapped against three available *C. nodosa* transcriptomes [Ruocco et al., 2017, Cymodocea nodosa Cn_1 Transcriptome (Project ID: 1264710) by Joint Genome Institute (JGI) and Dattolo et al. (*unpublished*)] by using the *blastn* algorithm (Altschul et al., 1997). Unambiguous positive-matches were determined if a percentage of identity ≥90% was found in stretches of sequences of at least 25 bp around the SNP position. This criterion for mapping was used to filter out possible errors that could result from non-specific similarities among homologous sites. Subsequently, the SNPs-carrying contigs were functionally annotated using the online Mercator-MapMan4 annotation tool (Schwacke et al., 2019).

## Results

### Accuracy of 2b-RAD Genotyping

**FIGURE 2:**
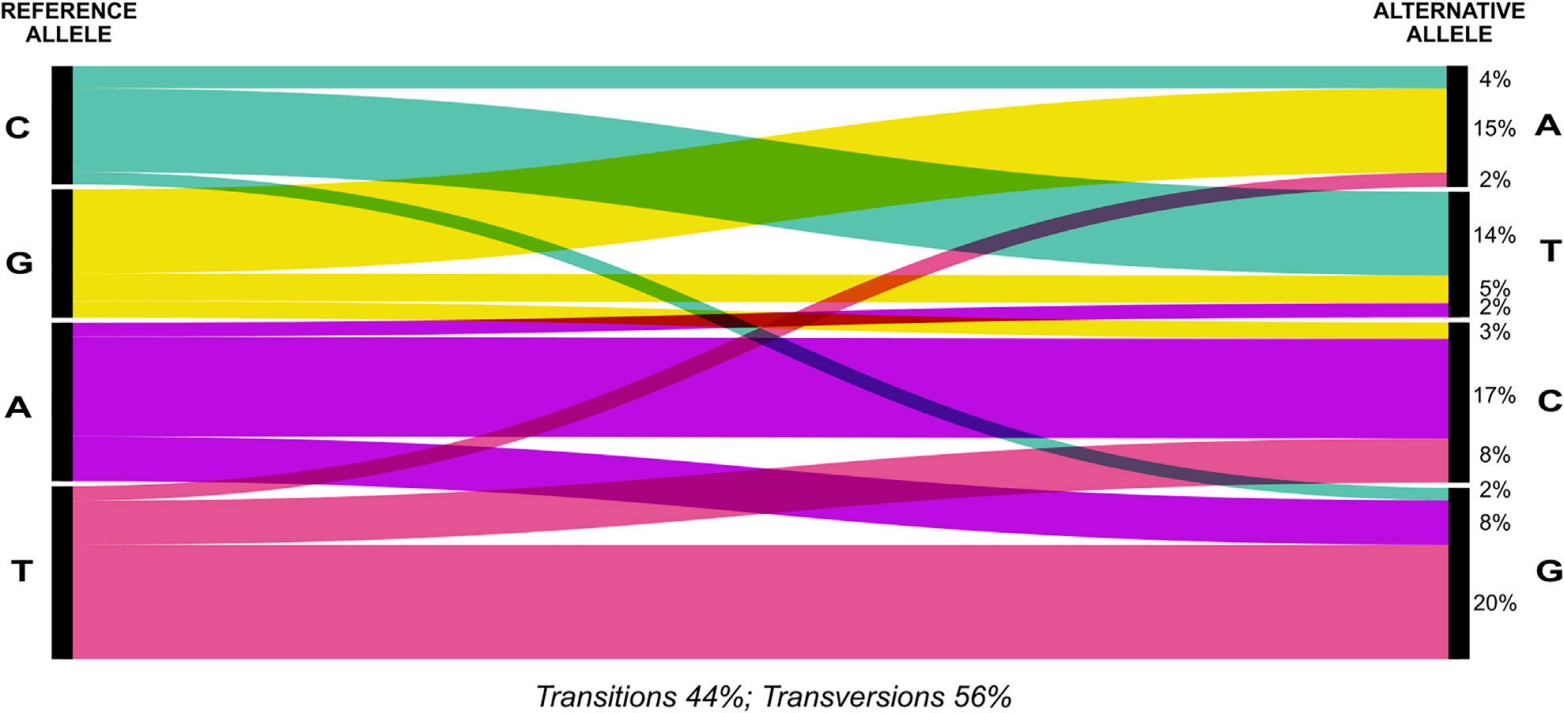
Distribution of transitions and transversions in the SNPs dataset. Percentages (%) of transitions and transversions of the whole set of 7,562 SNPs identified for 63 individuals is depicted using an alluvial diagram.

The 2b-RAD sequencing generated an average of 1,184,045.85 reads ± SD 425,645.65 reads per sample (Supplementary Table S2). Using a set of 2,100 SNPs that were reproducible across technical replicates as a training set for the VQSR, we estimated the (true) transition/transversion ratio as Ti/Tv = 1.61. When applying this recalibration we chose a tranche with 99% truth sensitivity as the cut-off to call all SNPs from the overall dataset. After filtering out seven poorly genotyped samples as well as highly heterozygous sites (possible lumped paralogous), we obtained a genotype dataset of 7,562 SNPs for 63 individuals (Supplementary Table S3). Overall, transversions were more frequent than transitions and composed 56% of the identified changes (Figure 2). The most frequent changes were G ↔ T (1857) and A ↔ G (1686) (Figure 2; Supplementary Table S4). Genotyping correspondences among technical replicated individuals was 89 ± 6% (Supplementary Table S5). For population differentiation analysis, our final dataset was further thinned to remove technical replicates, two samples that were clear outliers in an exploratory PCA and to only keep one SNP per RAD fragment with maximum allele frequency, resulting in 4,941 loci for 49 individuals.

### Population Differentiation, Genetic and Genotypic Diversity

**FIGURE 3:**
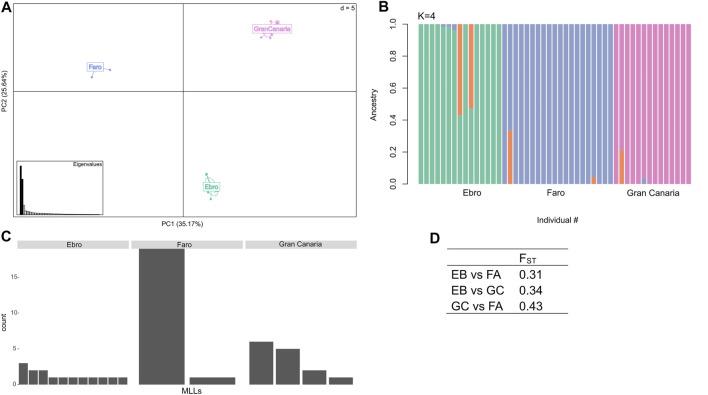
Population structure analysis and clonal diversity. **(A)** Principal Component Analysis (PCA) displaying the three *C. nodosa* populations collected along a latitudinal gradient of distribution (i.e., Gran Canaria, Faro and Ebro). **(B)** Admixture plot of the *C. nodosa* populations at K = 4. **(C)** Number of distinct Multi Locus Lineages (MLLs) identified at each sampling site. **(D)** Weir and Cockerham F_ST_ weighted estimate between populations.

Principal Component Analysis (PC1 = 35.2% and PC2 = 25.6% total variance; Figure 3A) showed a strong genetic differentiation among the three sampling sites along the latitudinal gradient and this was further confirmed by results from ADMIXTURE analysis (Figure 3B). In this analysis, K = 4 was identified as the “optimal K” with the lowest cross-validation error of 0.239 (Supplementary Table S6).

The three sampled populations had different levels of clonality. The *C. nodosa* population from Ebro Delta exhibited the highest genotypic richness (i.e., 11 distinct MLLs over 15 individuals, *R* = 0.71), in respect to Faro and Gran Canaria populations [i.e., only 2 (*R* = 0.05) and 4 (*R* = 0.23) distinct MLLs, respectively] (Figure 3C ,Table 1). In particular, in the *C. nodosa* population from Ria Formosa lagoon (Faro), one MLL largely dominated over the other (Figure 3C). To support clonal determination with SNP markers, we compared these data with those obtained for a subset of samples of the same populations using previously developed polymorphic SSR markers (Supplementary Table S7). Based on microsatellites, the *C. nodosa* population from Ebro Delta exhibited the highest genotypic richness with 11 MLGs over 12 samples (*R* = 0.91), while in Gran Canaria 6 distinct MLGs were identified (*R* = 0.45). In Faro, a single MLG was identified with microsatellite markers (Supplementary Table S8).

**TABLE 1:** Information on sampling sites, sample details and genetic indices obtained with 2b-RAD genotyping. For each population, the following information are shown: acronym, geographic coordinates (latitude and longitude), number of sequenced individuals N and number of additional technical replicates in parenthesis, number of individuals retained after filtering procedures and removal of technical replicates, total number of distinct Multi Locus Lineages (MLLs), *R* value (G-1/N-1), observed heterozygosity (H_o_) and inbreeding coefficient (F_IS_).

Sampling site	Acronym	Coordinates	Total N	N after filtering	MLLs	*R*	H_o_	F_IS_
Las Palmas de Gran Canaria—Canary Islands	GC	27°45′14″N, 15°40′18″W	18 (4)	14	4	0.23	0.13	−0.20
Ria Formosa lagoon—Faro, Portugal	FA	36°59′23″N, 7°50′01″W	20 (4)	20	2	0.05	0.24	−0.52
Ebro Delta, Spain	EB	40°34′48″N, 0°35′45″E	20 (4)	15	11	0.71	0.21	−0.13
Total	—	—	58 (12)	49	17	—	—	—

Genetic differentiation (based on allele frequencies across all loci) was significant for each population pair comparison (see exact G test in Supplementary Table S9). Global weighted pairwise F_ST_ values ranged from 0.31 to 0.43, with Faro and Gran Canaria exhibiting the highest differentiation (Figure 3D). Overall, inbreeding coefficient values (F_IS_) were negative for all populations, with the lowest value observed in Faro (Table 1). Mean observed heterozygosity (H_o_) was highest in Faro, intermediate in Ebro and lowest in Gran Canaria (Table 1). Values of genetic differentiation (exact G test), F_ST_, F_IS,_ and observed heterozygosity (H_o_) indicated above have been obtained following clones’ removal from the dataset. Genetic diversity and differentiation analyses repeated using SSR markers (Supplementary Table S8) gave comparable results across populations.

### Candidate Loci for Environmental Adaptation

BayeScan identified 9 F_ST_ outlier SNPs across all samples at q<0.3 and *P* >0.7 (Supplementary Figure S2A; Supplementary Table S10), while pcadapt identified 188 outliers based on PCA with a Bonferroni corrected *p*-value ≤0.001 (Supplementary Figure S2B,C; Supplementary Table S10). All 9 outliers identified by BayeScan were included among those identified by pcadapt (9 shared outliers; Supplementary Figure S2D). Interestingly, the frequency of the alternative allele for the 9-shared outliers was maximum in individuals from the Ebro population (Supplementary Table S11). The functional annotation of the 9-shared outlier SNPs is provided in Table 2, while full annotation of all outliers identified with one or both methods is provided in Supplementary Table S12.

**TABLE 2:** Functional annotations of the 9 significant outlier SNPs shared between BayeScan and pcadapt. SNPs ID; *E*-value; identity with %; bp of the query match; contig name (representing the best hit of the blast analysis against available transcriptomes^b^); name, description and related GO of the best hit of *Arabidopsis thaliana* according to TAIR (https://www.arabidopsis.org/index.jsp); bin codes of MapMan classification; and mutation type of the SNP (NS, nonsynonymous; SYN, synonymous), are outlined. Full annotation of all outlier SNPs obtained with one or both methods can be retrieved from Supplementary Table S12.

SNP ID	*E-*value	Identity (%)	Query match	Best hit *C. nodosa*	Best hit *A. thaliana*	Description TAIR	GO biological process TAIR	MapMan BIN	Mutation
chr1_673698^a^	—	—	—	No hits found	—	—	—	—	—
chr1_421195^a^	3E-10	36/37 (97)	(11:47)	TRINITY_DN164033_c0_g1_i1	AT3G13682	Lysine-specific histone demethylase 1 homolog 2. Involved in H3K4 methylation of target genes including the flowering loci FLC and FWA	Histone H3-K4 methylation	26.7	NS: Glu/Gly
chr1_555347^a^	3E-10	36/37 (97)	(31:67)	TRINITY_DN7669_c0_g1_i6	AT5G64060	NAC domain containing protein 103	Regulation of transcription, DNA-templated	27.3.27	NS: Ala/Thr
chr1_549579^a^	—	—	—	No hits found	—	—	—	—	—
chr2_19511^a^	8E-11	34/34 (100)	(7:40)	TRINITY_DN5447_c0_g1_i3	—	—	Not assigned, unknown	—	—
chr1_379730^a^	—	—	—	No hits found	—	—	-	—	—
chr2_1253^a^	0.005	27/29 (93)	(20:48)	TRINITY_DN226_c2_g1_i1	—	—	Not assigned, unknown	35.2	—
chr1_645789^a^	1E-9	35/36 (97)	(21:56)	TRINITY_DN7020_c1_g2_i3	AT5G04840	bZIP protein	Positive regulation of transcription, DNA-templated, regulation of transcription, DNA-templated	27.3.35	NS: Ala/Val
chr1_656786	—	—	—	No hits found	—	—	—	—	—

a Outlier SNPs, retained after clone removal by at least one method.

b Contig names assigned based on the *Cymodocea nodosa transcriptome* by JGI.

Five of the 9 shared outlier SNPs could be associated to specific contigs using available *C. nodosa* transcriptomes (Table 2). Two of these SNPs-carrying contigs (chr1_555347 and chr1_645789) encoded for proteins with a function in transcription regulation (“Regulation of transcription, DNA-templated”). One SNP (chr1_421195) was specifically associated to a gene involved in chromatin remodelling (“Histone H3-K4 methylation”) and photoperiodism/regulation of flowering time, namely *Lysine-specific histone demethylase 1 homolog 2* (Martignago et al., 2019; Table 2). All three annotated SNPs involved non-synonymous aminoacid substitutions (Table 2). The other two SNPs associated with specific contigs had unknown functions (Table 2). When the analysis of the outlier SNPs was repeated keeping only distinct MLLs for each population, 8 over the 9 shared-outliers were significantly retained by at least one method (pcadapt at *P* ≤ 0.01) (Table 2). BayeScan identified the same loci as “top outliers” but with a lower (ns) posterior probability *P* >0.3.

**FIGURE 4:**
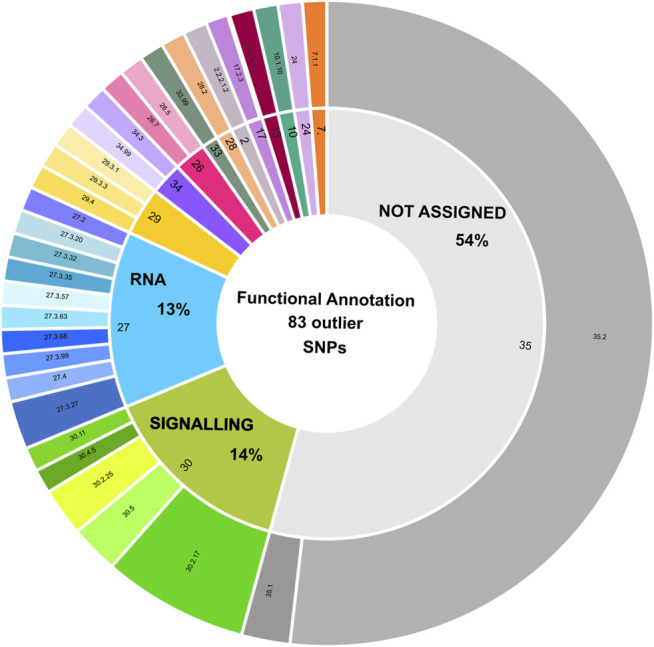
Functional annotation of 83 outlier loci displaying a positive match with transcribed regions. Functional annotation of SNPs with a positive blast hit against *C. nodosa* transcriptomes is depicted using a sunburst diagram. Each sequence has been assigned to a MapMan BIN category and sub-category based on its biological role or enzymatic activity. The definition of the BINs is included in the figure legend. A detailed description of BIN sub-categories can be retrieved from Supplementary Table S12.

Overall, 83 from the total of 188 outlier SNPs identified *via* Bayescan and/or pcadapt could be included in 14 different MapMan BINs (Figure 4; Supplementary Table S12). Among those, 27% SNPs-carrying contigs were associated to signalling (14%) and RNA metabolism (13%), while 54% of SNPs were not assigned (Figure 4). Within the “RNA” BIN (n. 27), 7 contigs encoded for transcription factors of various families (e.g., GARP, NAC, WRKY, bZIP) (Supplementary Table S12). In the “signalling” BIN (n. 30), 9 contigs were associated to receptor kinases (e.g., DUF 26), which play a critical role in plant response to stimuli and defence (Dievart et al., 2020), while 2 were wall associated kinases, which are involved in sensory and signal transduction pathways between the inner and outer surroundings of cell walls (Kohorn, 2001; Supplementary Table S12).

## Discussion

Populations distributed over broad latitudinal gradients often show patterns of clinal variation in phenotype and/or genotype (Hut and Beersma, 2011; De Frenne et al., 2013; Bergland et al., 2016; Machado et al., 2016). Through a genome-wide SNPs analysis based on 2b-RAD genotyping, we here demonstrated the presence of differentiated polymorphisms in three populations of the seagrass *C. nodosa* along a latitudinal gradient encompassing the Atlantic-Mediterranean transition region. Confirmed by two differentiation-based outlier tests, we identified 9 outlier SNPs, three of which could be reliably associated to genes involved in functions relevant to environmental adaptation. Notably, one SNP-carrying contig encoded for a protein with a key role in chromatin remodelling *via* histone methylation, and specifically linked to the regulation of photoperiodism and flowering time in land plants (He, 2009; Zhou et al., 2018; Martignago et al., 2019). This supports our initial hypothesis that specific genetic polymorphisms linked to important traits (e.g., flowering) could be present in *C. nodosa* inhabiting different latitudes possibly underlying local adaptation of populations to different photoperiod-temperature cues or other environmental parameters.

Our population genomic data, based on both SSRs and SNPs markers, showed a clear genetic differentiation among the three populations analysed. Besides being genetically differentiated, they also exhibited variable levels of genetic and genotypic diversity. Previous studies on the genetic structure of *C. nodosa* along its distributional range based on SSRs, revealed a strong genetic discontinuity between Atlantic and Mediterranean regions and a certain divergence between western and eastern Mediterranean (Alberto et al., 2008). Yet, a great heterogeneity was found across populations in terms of clonal and genetic diversity (Ruggiero, 2004; Alberto et al., 2006; Alberto et al., 2008; Máñez-Crespo et al., 2020). A trend for decreasing allelic richness from the eastern Mediterranean towards the Atlantic was also advocated to explain the low ability of Atlantic populations to resist disturbances (Tuya et al., 2021). Our results are in line with these findings, as the Gran Canaria population (GC), exhibited the lowest genetic diversity (as observed heterozygosity, H_o_) both with SSRs and SNPs markers. Genotypic richness (*R*) has been found to be highly variable across *C. nodosa* populations, even at small geographical scales (Alberto et al., 2006, 2008; Tuya et al., 2019). For instance, in the Canary Islands, the average *R* value previously calculated with available microsatellite markers was around 0.6, while it could vary from 0.3 to 0.9 for different meadows (Alberto et al., 2006; Máñez-Crespo et al., 2020). Genotypic richness obtained here for the Gran Canaria population with 7 SSR markers was within this range (0.4), while genotypic richness assessed with the SNP-dataset was lower (0.2). The *C. nodosa* population of the Ria Formosa lagoon (FA) has been historically considered monoclonal. Alberto et al., 2008 using SSR markers found only 5 genets over 220 ramets. Here using SSRs, we identified one single MLG over 18 samples analyzed, while using SNPs, 2 distinct MLLs could be detected. In general, we found a non-perfect match in the clonal discrimination power of SSRs and SNPs. SNPs have generally slower average mutation rates than SSRs; hence, each SNP is typically less informative (Allendorf, 2017). However, this is normally compensated by the much higher number of SNP markers, such that RAD-Seq can perform as well as, or better than SSRs in detecting population structure/divergence (Lemopoulos et al., 2019; Camacho-Sanchez et al., 2020; Sunde et al., 2020). However, SNP-datasets based on reduced representation libraries with moderate sequencing depth are also characterized by a substantial degree of missing data, error in SNP calling due to sequencing errors, lack of read depth or other sources of spurious allele calls (Mastretta-Yanes et al., 2015), which makes the designations of clones challenging (Kamvar et al., 2015). The challenge lies in particular in deciding on a threshold between designating a clone vs. a unique genotype. Several possible thresholds have been discussed (Kamvar et al., 2015), and we decided to use the difference between technical replicates as threshold for setting the limit we believe to be sequencing errors. While details differ slightly between SSRs and SNPs, general patterns are similar, and are also confirmed by other genetic parameters, particularly the inbreeding index F_IS_ that is influenced by clonality. All three populations exhibited strongly negative F_IS_ values based on the SNP genotyping, but the most clonal population (Faro) exhibited the lowest F_IS_ (-0.51). Negative F_IS_ has been shown to be strongly linked to the degree of clonality in facultative sexually reproducing species driven by genetic drift, both in theoretical (Prugnolle and De Meeûs, 2008) and population genetic assessments (Arnaud-Haond et al., 2020; Reynes et al., 2021b). Moreover, F_IS_ has been shown to be not as sampling-sensitive as genotypic richness *(R)* and has been suggested as the measure of choice for assessing the importance of clonal reproduction in seagrasses (Arnaud-Haond et al., 2020).

While SNPs may have their challenges in clone detection, one of the main advantages of reduced representation sequencing is the possibility to identify locus-specific polymorphisms potentially responsible for local adaptation of populations to specific environmental conditions (Zimmerman et al., 2020). Here, we identified nine SNPs potentially involved in environmental adaptation of *C. nodosa* populations along its latitudinal distribution (Table 2). These nine outlier loci could either be under selection themselves, or they could be linked to loci involved in local adaptation. The high levels of linkage disequilibrium generated by the lack of recombination in clonal populations could conserve such linkage between neutral and selected sites to a higher degree than in sexually reproducing species. However, the consequences of clonal reproduction on genomic architecture and the effects on the theory of local adaptation are so far little investigated. The three loci with a functional annotation encoded for proteins with a role in regulation of transcription. In all three loci, the mutation at the protein level was non-synonymous, hence providing a change in the aminoacid sequence. Interestingly, when considering the functional annotation of the whole outliers’ dataset (Supplementary Table S12), the biological processes “signalling” and “regulation of transcription” constituted a large part of those associated with our sequences. In total, nine transcription factors (TF) of different families (e.g., NAC domain-containing TFs, WRKY, bZIP, and JUMONJI), were found across the outlier-dataset. Similarly, a number of cysteine-rich receptor-like protein kinases, cell wall-associated ser/thr kinases, and inositol 1,3,4-trisphosphate 5/6-kinase family proteins, were identified. This highlights the fundamental role signal integration and gene-regulatory networks could play for enabling adaptation to environmental conditions, as they allow to coordinate extracellular signals and intracellular regulatory machinery (Lopez-Maury et al., 2008).

Notably, one of the 9-shared outlier SNPs was specifically associated with photoperiodism and regulation of flowering time (chr1_421195), which is in an ecologically important life history trait. The timing of floral transition is critical to reproductive success in plants and varies across latitudes in response to changes in photoperiod and temperature patterns (Bäurle and Dean, 2006; Wilczek et al., 2010; Brambilla and Fornara, 2013). In *Arabidopsis*, this trait is genetically controlled by a network of flowering genes, whose expression is regulated by various chromatin modifications, among which is a central regulator of flowering*, FLOWERING LOCUS C* (FLC) (He, 2009; Wilczek et al., 2010). FLC inhibits floral transition largely by reducing the expression of flowering-time integrators, such as *SUPPRESSOR OF OVEREXPRESSION OF CONSTANS 1* (SOC1) and *FLOWERING LOCUS T* (FT) (He et al., 2003; He, 2009). The regulation of FLC expression is associated with various “active” chromatin modifications including histone H3 lysine-4 (H3K4) methylation and various “repressive” histone modifications, as H3K4 demethylation and H3 lysine-27 (H3K27) methylation (He, 2009). Chr1_421195 was included in a contig encoding for *Lysine-specific histone demethylase 1 homolog 2* (LDL2)*.* In *Arabidopsis*, this gene is involved in H3K4 (de)methylation of target genes including FLC. FLC is down-regulated by LDL2 and ldl2 mutants display increased H3K4me3 levels at FLC compared to wild type (Martignago et al., 2019). LDL2 is additionally involved in the control of H3K4 methylation state of FWA, a homeodomain-containing transcription factor which interferes with floral transition (Jiang et al., 2007) and controls primary seed dormancy related-genes (Zhao et al., 2015). A similar function in regulating the histone methylation pattern of flowering genes has been also suggested for LDL homologs in other plant species (Gu et al., 2016). The mutation we identified for this locus in *C. nodosa* populations was non-synonymous, leading to a substitution of a Glutamic acid (Glu) in Glycine (Gly). We cannot establish the real effect of this substitution at the protein level, but as it will modify the charge of the peptide, it could potentially result in a loss of interactions with other molecules or could induce differences in the regulation of other genes related to the flowering pattern (Caicedo et al., 2004). It is worth noticing that the frequency of the alternative alleles of the locus chr1_421195 was maximum in individuals from the Ebro population (Supplementary Table S11), whereas the reference allele was present in Faro and Gran Canaria. Although we expected this locus to diverge in populations exposed to large differences in photoperiod, this was not the case as e.g. Faro and Ebro had the strongest similarity in daylight and seasonality variations among sampling sites. However, studies in terrestrial plants have demonstrated that even small differences in the duration of the light period might have large effects on flowering time (Roden et al., 2002). In addition, the strong level of the differentiation at this locus across populations could also be related to differences in the temperature regimes rather than photoperiodic cues. In *Arabidopsis*, vernalization-induced flowering is mediated by epigenetic regulations of FLC (Sharma et al., 2020); while other studies indicated several genes belonging to the *FLOWERING LOCUS* family influence flowering time in responses to the ambient growth temperature (Posé et al., 2013; Capovilla et al., 2015). We cannot exclude that this polymorphism could also be linked to differences in other environmental settings across sites or to a differential regulation of specific biological processes among the analysed populations.

Across the whole dataset of outlier SNPs (Supplementary Table S12), other loci with a functional annotation, were involved in the regulation of flowering time and circadian rhythms. Chr1_659852 was associated to a contig encoding for *PWWP domain protein 3*, which is specifically involved in the regulation of FLC and flowering time. PWWP domain proteins (PDPs) function together with other components (e.g., *FLOWERING LOCUS VE* and *MULTICOPY SUPPRESSOR OF IRA 5*) to regulate the function of the PRC2 histone methyltransferase complex, thereby facilitating the maintenance of H3K27me3 on FLC (Zhou et al., 2018). In addition, other two SNPs-carrying contigs (chr1_546361 and chr1_474761) encoded for transcription factors involved in flowering regulation and response to temperature stimulus. The first encoded for the protein JMJD5/JMJ30, which contains a jumonji-C (jmjC) domain, the second for *EARLY FLOWERING MYB PROTEIN* (EFM). In *Arabidopsis*, JMJD5 has a histone demethylase activity (H3-K36 specific) and interacts with EFM to repress the floral pathway integrator FT, thus negatively regulating flowering (Yan et al., 2014). In addition, EFM participate in the flowering thermosensory pathway of *Arabidopsis* (Gan et al., 2014).

Taken together, our results highlight that polymorphisms at flowering-related loci and transcription factors could make an important contribution to genetic adaptation of *C. nodosa* across latitude, although this should be further confirmed by larger-scale studies and *ad hoc* common-garden or reciprocal transplantation experiments (Reusch et al., 2014; Pazzaglia et al., 2021). Flowering-regulating loci, integrating annual responses to light and temperature patterns through complex gene-regulatory networks, determine the timing of reproduction, which is crucial for fitness and survival of populations (Austen et al., 2017), besides the plasticity of single long-lasting genotypes (Pazzaglia et al., 2021). This could also contribute to differences in flowering phenology observed in this species along the depth gradient (Buia and Mazzella, 1991) or across regions (Máñez-Crespo et al., 2020). Yet, epigenetic modifications, such as histone modifications, seem to play an important role in the regulation of flowering-related gene networks, and potentially other phenology-related pathways, in *C. nodosa*. In support of this, a recent work has demonstrated different levels of global genomic cytosine methylation (5-mC) in *C. nodosa* populations from contrasting thermal environments, as well as a low level of gene body methylation (predicted by high CpG_O/E_ ratio) in transcripts with a differential expression depending on plants’ origin (Entrambasaguas et al., 2021).

While we acknowledge that our study including only three *C. nodosa* populations with a high level of clonality in at least two of them, has limitations, it sheds first light on genetic polymorphisms and related biological processes that could contribute to environmental adaptation of *C. nodosa* and potentially other seagrass species across their biogeographic range of distribution. In addition, we are aware that, although genome scan methods have been often applied in partially clonal organisms, there is a general lack of predictive works about the power of such methods in case of very strong clonality rate, as in our case. Future seascape genomic studies based on upgraded whole-genome information and employing a larger number of populations (or species) along latitudinal clines would allow a higher power for detecting signature of selection associated to local environmental settings in seagrasses.

## Data Availability

The raw Illumina sequences used in the study are available in the Sequence Read Archive (SRA) repository under NCBI Bioproject PRJNA814006. Vcf files are available as Supplementary Material (Data Sheet 2).
