# Supplementary figures and images for: 2b-RAD Genotyping of the Seagrass Cymodocea nodosa Along a Latitudinal Cline Identifies Candidate Genes for Environmental Adaptation
Source: Front Genet. 2022 May 16;13:866758. doi: 10.3389/fgene.2022.866758 (PMC9149362; doi:10.3389/fgene.2022.866758)

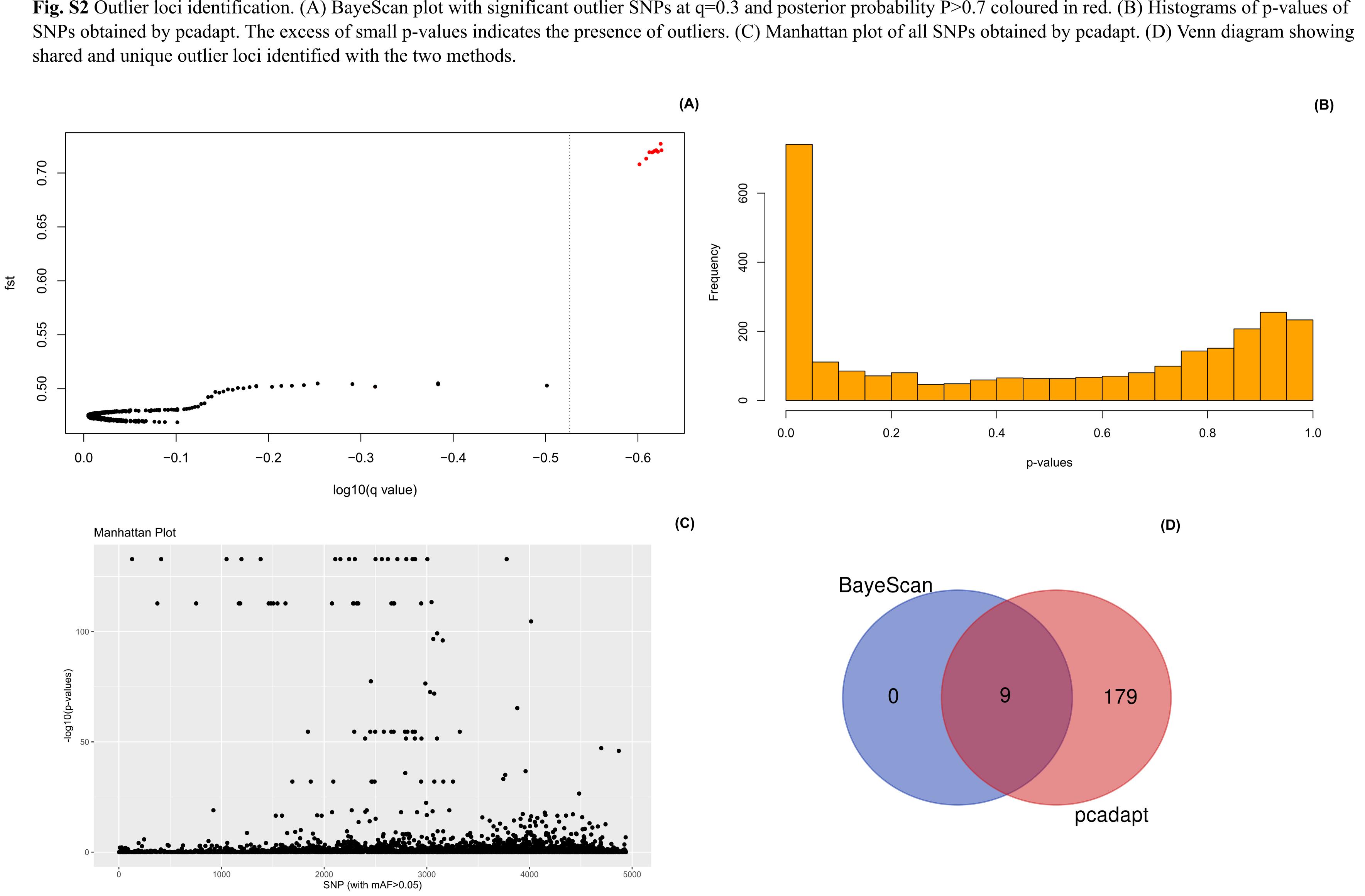

Supplement: Supplementary file 1 [file Image2.PNG]

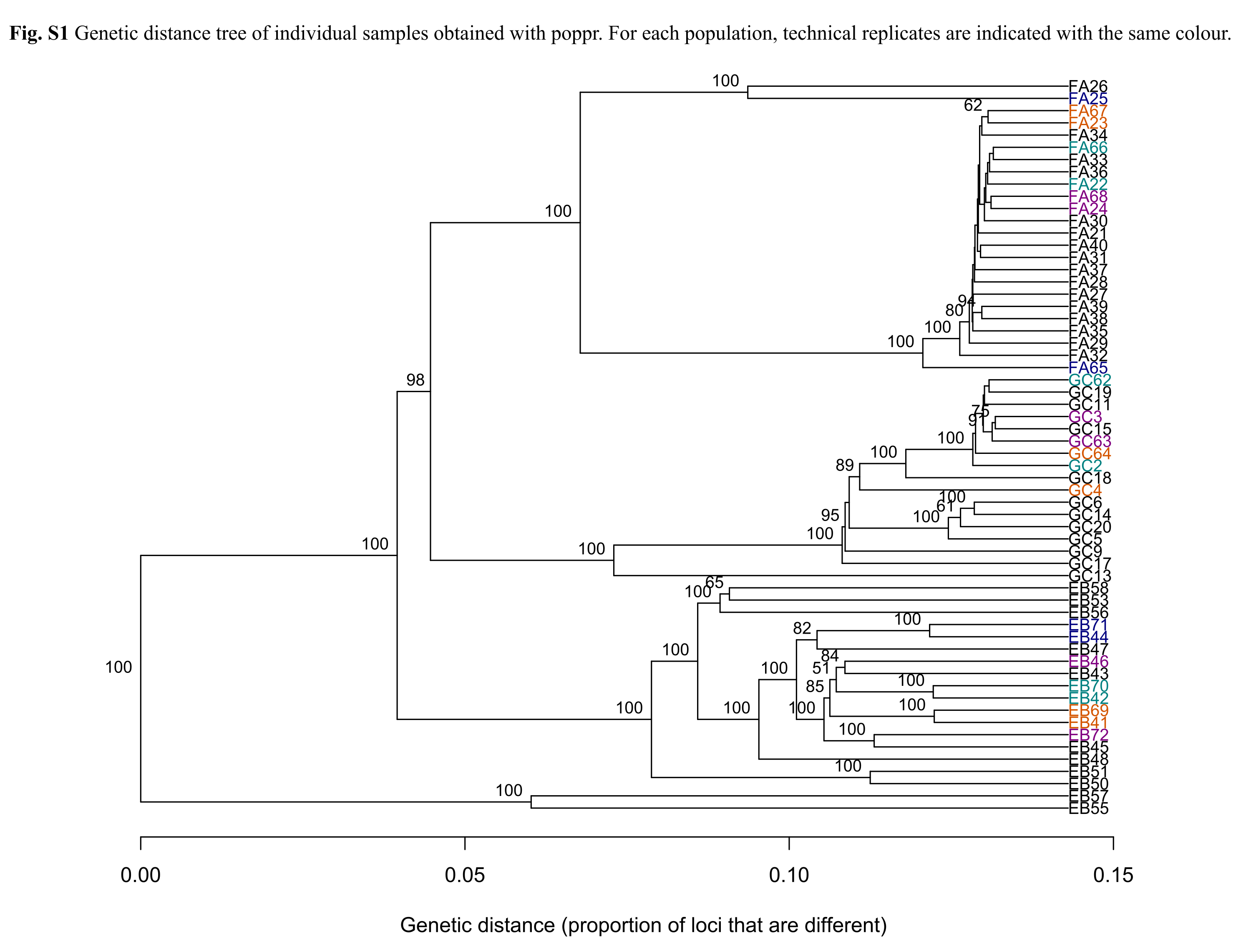

Supplement: Supplementary file 2 [file Image1.PNG]
